# From 'ICDAS' to 'CariesCare International': the 20-year journey building international consensus to take caries evidence into clinical practice

**DOI:** 10.1038/s41415-021-3732-2

**Published:** 2021-12-17

**Authors:** Nigel B. Pitts, Avijit Banerjee, Marco E. Mazevet, Guy Goffin, Stefania Martignon

**Affiliations:** 41415112889001 Faculty of Dentistry, Oral & Craniofacial Sciences, King´s College London, Tower Wing, Guy´s Hospital, London, SE1 9RT, UK; 41415112889002 UNICA – Caries Research Unit, Research Department, Universidad El Bosque, Bogotá, Colombia

## Abstract

This paper charts the 20-year collaborative journey made by international teams of dental researchers, educators and practitioners. Following the initial development of the International Caries Detection and Assessment System (ICDAS) in 2002, the International Caries Classification and Management System (ICCMS) was collaboratively developed between 2010-2017 with several dental research and practice organisations, and influenced by best evidence judged via SIGN methodology, the UNEP Minamata Treaty (and linked phasing down of dental amalgam), three Dental Policy Labs and an international movement in operative dentistry to move towards minimally invasive dentistry. The FDI World Dental Federation publicised and advocated the ICCMS in 2019, when the 'CariesCare International' Consensus Guide and 4D caries management system was published to aid the delivery of ICCMS into practice. This system, which is designed to help practitioners deliver optimal caries care for patients, is now being adapted internationally for post-pandemic use in the 'Caries OUT' study. It is also being used as a vehicle for implementing the updated *Delivering better oral health* guidance on caries, as part of the minimum intervention oral healthcare delivery framework in the UK.

## Key points

- The International Caries Detection and Assessment System (ICDAS) has synthesised and harmonised international evidence on caries detection and assessment in order to enable better integration/communication of knowledge across caries research, education, public health and clinical practice.
- The International Caries Classification and Management System (ICCMS) has built on the ICDAS to provide an international consensus on comprehensive, risk-based, minimally invasive and preventively orientated caries management.
- CariesCare International 4D Caries Management has again been built through international consensus on how best to deliver ICCMS in dental practice and is now being adapted internationally for post-pandemic use and for implementing the updated *Delivering better oral health* guidance on caries, as part of the minimum intervention oral healthcare delivery framework, in the UK.

## Introduction

Although there has been an evidence base around the dental 'caries continuum', a process starting with the initial lesion confined to dental enamel and gradually increasing in severity to more advanced stages of clinical cavitation, since the late 1800s, through the research and teachings of G. V. Black in particular, this understanding was not widely disseminated or understood. Structured and reliable caries classification systems including 'initial' or 'non-cavitated lesions' in enamel have been devised for research and published throughout the 1950s, with Backer Dirks' seminal work from the Netherlands;^1^ the 1960s, Marthaler's Swiss system;^2^ the 1980s, with Pitts and Fyffe^3^ reporting studies in Hong Kong; and in the 1990s, with Ismail and co-workers^4^ in Canada. Ekstrand *et al.*^5^ from the UK and Denmark added in gold-standard lesion staging validity and lesion activity, as well as Nyvad *et al.*,^6^ also from Denmark, focused on caries activity assessment. However, much of this knowledge and evidence remained in research silos and was not generally recognised in many countries across the wider domains of dental education, public health and clinical practice.

Traditionally, caries has been managed operatively by ever more rapid forms of 'drilling and filling', leading to the destructive 'repeat restorative spiral' demonstrated by Elderton^7^ in Scotland in the 1990s and subsequently shown be to an unwelcome and prevalent phenomenon in many countries. The lack of an underpinning preventive philosophy was often combined with wide variations in personalised care planning between different dentists, even when examining the same patients.^7^

## The initial development of 'ICDAS'

This paper charts the collaborative journey made since 2001 by international teams of dental researchers, educators and practitioners seeking to synthesise and harmonise the international evidence on caries detection, assessment and management to enable better communication of knowledge across the four domains of epidemiology, practice, research and education.

Variations in the way caries was defined, detected and classified were a continuing concern in the 1990s. This became painfully obvious at a pivotal International Consensus Workshop on Caries Clinical Trials,^8^ which occurred, delayed, in January 2002. To the embarrassment of the international research and practice community and organisations, as well as the dental industry and regulators present, it was found that attempts at comparing results from different trials in a systematic way were compromised by the bewildering array of incompatible criteria and diagnostic systems used to detect, assess and record caries. Several of the lead participants from the workshop vowed to address the issue by sharing information and expertise internationally to harmonise diagnostic criteria based on best underpinning evidence. A key consensus recommendation from this workshop was to aid future clarity by defining out three key aspects:^8^

- Lesion detection (which implies an objective method of determining whether or not disease is present)
- Lesion assessment (which aims to characterise or monitor a lesion, once it has been detected)
- Caries diagnosis (which should imply a human professional summation of all available data).

**Fig. 1:**
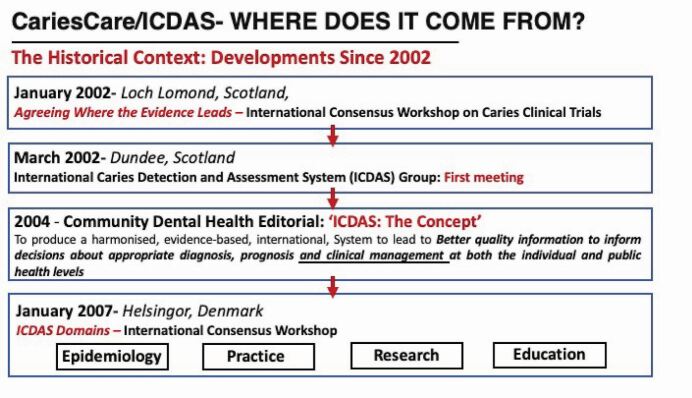
The historical context

This development group accepted the need to separate out lesion detection and assessment when it first met in March 2002 to start work on developing an International Caries Detection and Assessment System (ICDAS) (Fig. 1). This led to the formation of the ICDAS Foundation, which became a UK-registered charity. The concept was to produce a harmonised, evidence-based, international system to lead to 'better quality information to inform decisions about appropriate diagnosis, prognosis and clinical management at both the individual and public health levels'.^9^ As depicted graphically by the ICDAS 'wardrobe', a common system was designed to be suitable for use across epidemiology, practice, research and education. Published in 2004, the system was developed to facilitate appropriate clinical caries management due to continuing concerns that, despite the evidence, many countries were still not ready to 'move from operative to non-operative/preventive treatment of dental caries in clinical practice'.^10^

The ICDAS Board has, from the start, involved representatives from Dundee University, subsequently King's College London, UK (Pitts); Michigan University, subsequently Temple University, US (Ismail); Indiana University, US (Zero) and Copenhagen University, Denmark (Ekstrand) as well as representatives of the FDI World Dental Federation and the National Institute of Dental and Craniofacial Research, US. In addition, a coordinating committee formed with key participation from Gail Douglas and Stefania Martignon.

The ICDAS methodology and approach has become accepted on a global basis, with researchers, educators, clinicians and public health dentists seeing the value of an internationally harmonised common approach to aid in the synthesis and dissemination of research findings as well as in teaching and clinical care. In terms of the quality and utility of the ICDAS work being recognised and cited by international peers in the field, it is helpful that a recent paper in the journal *Caries Research* presented a bibliometric analysis on the 'Top 100 papers in cariology'. Pleasingly, two key papers from the ICDAS Group in 2007^11^^,^^12^ were included in the top six of the top 100 most cited papers in cariology.^13^ In general comparisons across all time, the *Lancet* paper 'Dental caries' by Selwitz, Ismail and Pitts^11^ was ranked fourth while a *Community Dentistry and Oral Epidemiology* paper on the ICDAS^12^ by Ismail, Sohn, Tellez, Amaya, Sen, Hasson and Pitts was ranked sixth. When citations were considered by five-year period, by year, or by six-month period, the *Lancet* paper^11^ took first position in each case as the most cited paper.^13^

## Building international consensus to create and improve the 'ICCMS'

Having made progress with the basic elements of caries lesion detection and assessment, the teams involved with the ICDAS Foundation set out to broaden the scope of the systems and tools beyond these foundational aspects and move to include all the elements required for comprehensive, tooth-preserving, preventive caries management at both the person/patient level as well as the tooth level. To do this, collaborations were broadened and a wider circle of like-minded individuals and organisations joined the challenge to develop the ICCMS, all on a voluntary and unfunded basis.

In the education domain, many individuals from the ICDAS Foundation were also part of a joint initiative from the European Organisation for Caries Research (ORCA) and the Association for Dental Education in Europe (ADEE) to develop a standard core cariology curriculum. As part of this exercise, the working group tasked with developing the 'caries risk assessment, diagnosis and synthesis' elements integrated the ICCMS.^14^ Undergraduate cariology teaching consensuses were also facilitated in Colombia, US and four Caribbean countries. In the research domain, ICCMS became part of the activities of the International Association for Dental Research (IADR) *Global oral heath inequalities* Dental Caries Task Group.^15^ The team also continued to work with the FDI World Dental Federation.

**Fig. 2:**
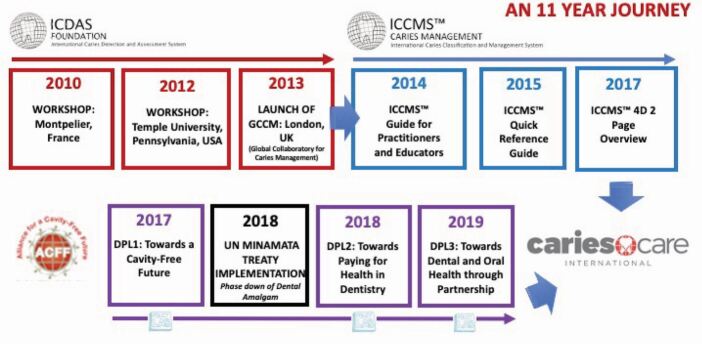
Phasing up caries prevention and MI management with ICCMS, logos reproduced with permission from ACFF, ICDAS Foundation and CariesCare International

The ICCMS was developed between 2010-2017, with refinements continuing to 2021. Figure 2 summarises key elements in 'Phasing up caries prevention and minimally invasive (MI) management with ICCMS', an 11-year journey to date. In 2010, an international workshop convened in Montpellier, France which agreed the specification of the key elements of the system. In 2012, a further workshop followed at Temple University at which the ICCMS was presented^16^ and international consensus was sought on caries management pathways to preserve dental tissues and promote oral health.^17^ The outcomes of these meetings led to the joint planning and delivery of a participatory four-day workshop to launch the Global Collaboratory for Caries Management (GCCM) at King's College London in the summer of 2013. This workshop, planned with implementation scientists, brought together a broad range of 75 dental researchers, educators and clinicians from 20 countries to use Scottish Intercollegiate Guidelines Network (SIGN) methodology to start using best evidence to map out the *ICCMS guide for practitioners and educators.*^18^ This proved to be a significant undertaking and sub-groups continued the guidance development and considered judgement and consensus processes over the next 18 months, with the guide being published in December 2014 as a 40-page document with 40 pages of appendices. The ICCMS guide was based on an updated version of the earlier International ICDAS Consensus for 'detection and assessment', but now included caries classification and risk assessment, a full synthesis with both care planning and delivery steps, followed by risk-based recall. Feedback on the guide from the 75 co-creators and from wider groups was positive, but although the group had decided that they wanted a comprehensive and detailed document, it was appreciated that an additional digestible (14-page) A5 size quick reference guide was needed, produced in 2015. A further breakthrough step in the implementation journey was the development of a two-sided sheet containing all the key information in a '4Ds' format in 2017. These materials are available from the ICCMS website (https://www.iccms-web.com/).

The lower row of Figure 2 shows parallel activities, mainly coordinated through the Alliance for a Cavity-Free Future (ACFF) (https://www.acffglobal.org/). During this period, there has been a growing awareness of the impact of the now ratified UNEP 'Minamata Treaty'^19^ and the consequent phasing down (in some countries phasing out) of the use of dental amalgam. This has helped many stakeholders appreciate the value of phasing up caries prevention, particularly where there are also environmental challenges associated with other restorative biomaterials, nanoparticles and plastics. Between 2017-2020, three ACFF/King's College London 'Dental Policy Labs' have been held, with different mixes of key stakeholders developing new ways to accelerate a policy shift towards increased resource allocation for caries prevention and control,^20^ creating and implementing acceptable prevention-based dental payment systems,^21^ and partnerships to enable positive behaviour in caries prevention and control among patients and the public.^22^ ICCMS played a part in all of these policy labs, incrementally helping to shape and refine a specific 'practice-friendly' format which ultimately became the CariesCare International '4D' system.

The other development evolving over this period and discussed at the Dental Policy Labs was the international move in operative dentistry towards a minimally invasive (MI) approach.^23^ This is one of the four interlinked clinical domains of the minimum intervention oral healthcare (MIOC) framework that forms the basis of patient-focused, modern oral healthcare delivery by all members of the oral healthcare team, across the disciplines of restorative dentistry, including cariology and caries management. The team have also developed validated questionnaires to explore dentists' motivation, capability and access to the resources needed to practise MI caries care with ICCMS.^24^

Over this period, there were developments with ICCMS being advanced as an example of a 'caries management pathway',^25^ followed at an international level by the FDI publicising ICCMS through a white paper in 2016,^26^ and specifically advocating the ICCMS and its philosophy to National Dental Associations in 2019 through an *FDI policy statement* on caries.^27^ This also led to the International Standards Organisation (ISO) using ICCMS terminology as the global standard for dental caries (https://www.iso.org/obp/ui/#iso:std:iso:1942:ed-3:v1:en).

As with the earlier ICDAS publications, there has been uptake of both the ICDAS and ICCMS over this later period and the systems have been cited in at least 423 research publications from 36 countries between 2014-2020. The systems have met the original aim by harmonising the definitions and conventions used for caries detection, assessment and clinical management across caries research, education, public health and clinical practice. Impact on terminology used in the field is evidenced by the recent international consensus recommendations.^28^

## Adapting ICCMS to take caries evidence into clinical practice with the 'CariesCare International' 4D caries management system

CariesCare International (CCI) 4D is essentially the practice version of ICCMS, delivered by a sub-branch of the ICDAS charity, CariesCare International. The objective is to improve caries prevention and control at the patient level by implementing ICCMS worldwide. There are shared goals for both ICCMS and CCI:

- To prevent new lesions from appearing (primary prevention)
- To prevent existing lesions from advancing further (secondary/tertiary prevention)
- To preserve tooth structure through appropriate:
  - Non-operative care
  - Conservative operative care
  - Managing risk factors, monitoring and reviewing.

The system is designed to deliver optimal caries care with and for patients.

Following the formation of CCI and its Executive (SM, NBP, MM and GG), the 2018 launch in Copenhagen set out the objectives and work started to produce the resources now available:

- The 2019 CariesCare practice guide: consensus on evidence into practice^29^ (44 authors from 20 countries)
- The 2019 caries classification and management in the context of the CCI consensus: a clinical case study^30^
- A dedicated website (https://cariescareinternational.com/) linked to social media with free downloadable resources including e-learning in both English and Spanish.

The reaction to CCI and website traffic has been positive to date in Europe, the US and Latin America. The concept of a version adapted for everyday practice which retains the key features of the full ICCMS has been welcomed and is seen as an enabling tool in the move towards effective clinical prevention.^31^

**Fig. 3:**
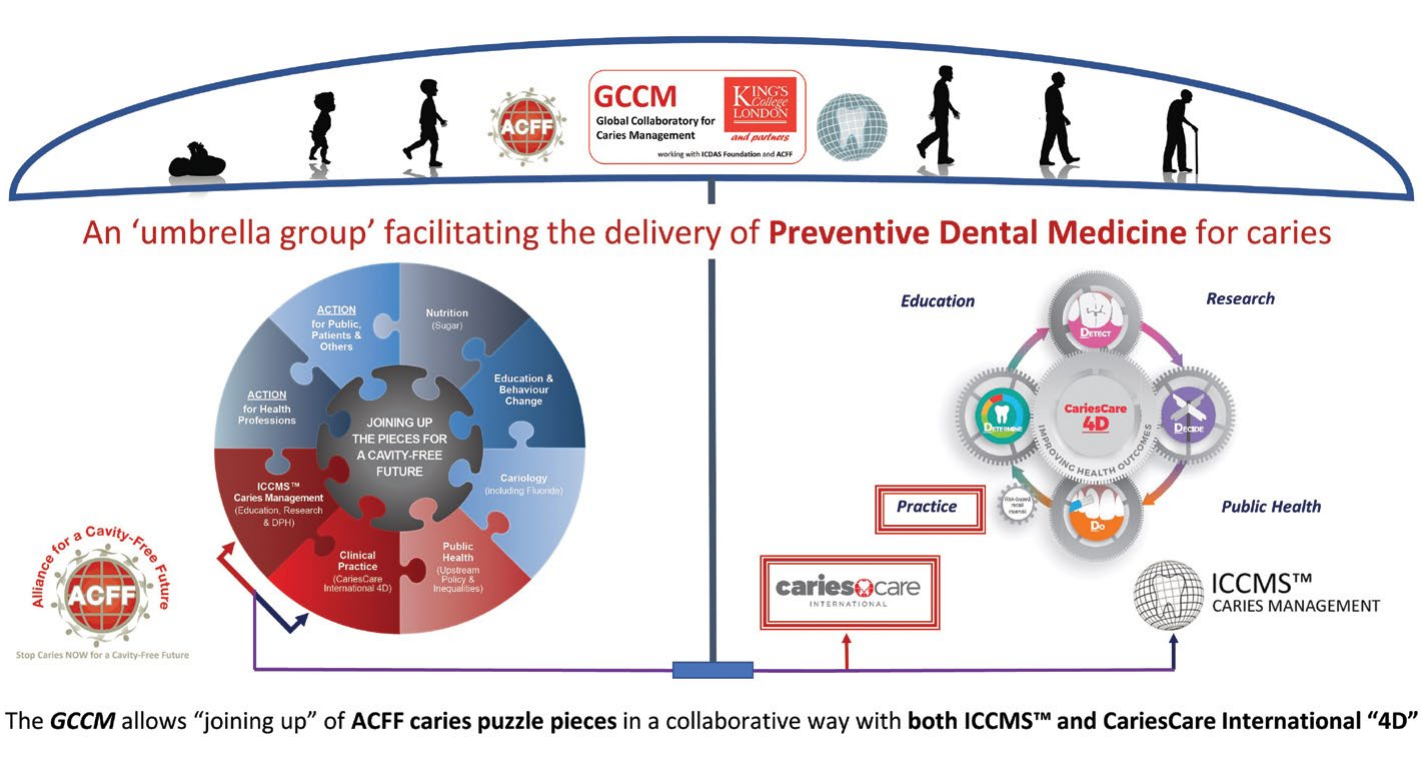
The Global Collaboratory for Caries Management, logo and illustrations reproduced with permission from ACFF; logos reproduced with permission from ICDAS Foundation, CariesCare International and King's College London

Figure 3 sets out an overview of how the GCCM has now evolved to help control tooth decay across the life course. The GCCM now facilitates the joining up of the ACFF 'caries puzzle' pieces in a collaborative way with both ICCMS and CCI '4D', contributing to securing a cavity-free future and moving towards the updated ICDAS Foundation vision of improving human health worldwide by the prevention and control of dental caries throughout life. CCI has also been fully adopted by the French Government as the model for preventive care in cariology in its pilot for a new payment system.

## Next steps in the journey

**Fig. 4:**
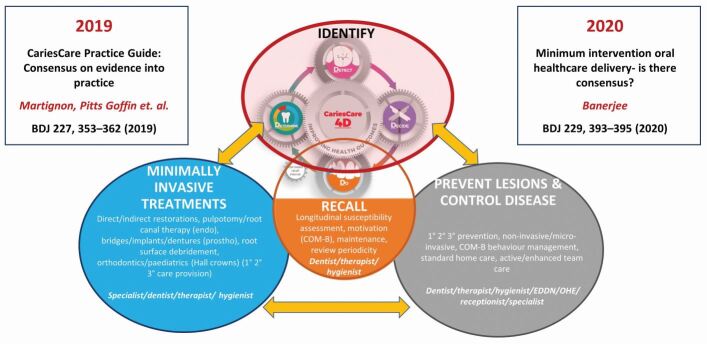
Using the CariesCare 4D model within the MIOC framework for delivering better oral health

This CCI 4D approach is also advancing in two new areas:

- Led by work in Colombia, international consensus for an oral health record is being built around the CCI 4D model and linked to the development of standardised software. This should facilitate adoption in both education and practice and will aid future research
- Following the impact of the COVID-19 pandemic, there has been a growing realisation that preventive caries management not only reduces the disease burden at the patient level but also reduces the need for aerosol generating procedures/exposures (AGPs/AGEs).^32^ Although the contamination potential of these AGPs/AGEs with proper infection control measures is still debated in the literature as this paper is being submitted, this situation has shown to professionals that these approaches are both compatible and desirable with their daily practice. An international group within CCI has been adapting CCI 4D protocols for post-pandemic use in the 'Caries OUT' study.^33^ Colleagues from 21 centres in 13 countries have been working throughout the pandemic, using teledentistry and a range of new approaches, in order to undertake a 12-month oral health outcomes study with CCI with no AGPs, as a multi-centre pragmatic clinical development study in schoolchildren. These developments fit well with a series of policy recommendations for 'making cavities history'^34^ from an international ACFF Taskforce and are in accord with the 2021 WHO Resolution on Oral Health which seeks 'to reorient the traditional curative approach, which is basically pathogenic, and move towards a preventive promotional approach with risk identification for timely, comprehensive and inclusive care'^35^
- With the creation of a new UK Chapter of the ACFF, plans are being developed to use the CCI 4D model as a vehicle for implementing the updated *Delivering better oral health* guidance (version 4) on caries management in the UK. This will be achieved (Fig. 4) by integrating CCI 4D within the four interlinked clinical domains (identification of disease, prevention/control, MI operative intervention and recall) of the oral healthcare team-delivered, patient-focused MIOC framework,^36^ as an exemplar for caries management in clinical practice.^23^^,^^37^^,^^38^^,^^39^^,^^40^

It should be appreciated that as has been made clear since the ICDAS was established, the work undertaken within this journey was made possible by the contributions of farsighted individuals across the preceding decades.^1^^,^^2^^,^^3^^,^^4^^,^^6^^,^^41^^,^^42^^,^^43^^,^^44^^,^^45^

## Conclusion

Following the development, refinement and iterative implementation of the ICDAS, ICCMS and CariesCare International methods and systems for dentists and their oral healthcare teams delivering clinical caries care, greater consistency and adoption of standards can be seen both by individuals and organisations. In addition, beneficial changes are now visible in the detection, assessment and management of caries in many countries across the domains of caries research, education, public health and clinical practice. In the caries world, this journey has provided the MI tools, based on best evidence and international consensus, to now 'Build back better, fairer and greener' - after the pandemic.
